# Exploring weight bias internalization in pregnancy

**DOI:** 10.1186/s12884-022-04940-4

**Published:** 2022-07-29

**Authors:** Taniya S. Nagpal, Ximena Ramos Salas, Michael Vallis, Helena Piccinini-Vallis, Angela S Alberga, Rhonda C Bell, Danilo F da Silva, Margie H Davenport, Laura Gaudet, Angela C Incollingo Rodriguez, Rebecca H Liu, Maxine Myre, Kara Nerenberg, Sarah Nutter, Shelly Russell-Mayhew, Sara C S Souza, Candace Vilhan, Kristi B Adamo

**Affiliations:** 1 Department of Kinesiology, Faculty of Applied Health Sciences, Brock University, St. Catharines, ON Canada; 2 Faculty of Kinesiology, Sport, and Recreation, University of Alberta, Edmonton, Canada; 3 Obesity Canada, Edmonton, AB Canada; 4 Department of Family Medicine, Dalhousie University, Halifax, NS Canada; 5 Department of Health, Kinesiology & Applied Physiology, Concordia University, Montreal, QC Canada; 6 Department of Agricultural, Food and Nutritional Sciences, Faculty of ALES, University of Alberta, Edmonton, AB Canada; 7 School of Human Kinetics, Faculty of Health Sciences, University of Ottawa, Ottawa, ON Canada; 8 Department of Obstetrics and Gynecology, Queen’s University, Kingston, ON Canada; 9 Psychological & Cognitive Sciences, Social Science and Policy Studies, Worcester Polytechnic Institute, Worcester, MA USA; 10 Women’s College Hospital Institute for Health System Solutions and Virtual Care (WIHV), Toronto, ON Canada; 11 Werklund School of Education, University of Calgary, Calgary, AB Canada; 12 Department of Medicine, Cumming School of Medicine, University of Calgary, Calgary, AB Canada; 13 Department of Educational Psychology and Leadership Studies, University of Victoria, Victoria, BC Canada; 14 Obesity Canada, Patient Advocate Volunteer, Edmonton, AB Canada

**Keywords:** Pregnancy, Weight bias, Obesity, Maternal health, Stigma

## Abstract

**Background:**

Recent research has shown that pregnant individuals experience weight stigma throughout gestation, including negative comments and judgement associated with gestational weight gain (GWG). Weight bias internalization (WBI) is often a result of exposure to weight stigma and is detrimental to biopsychological health outcomes. The purpose of this study was to explore WBI in pregnancy and compare scores based on maternal weight-related factors including pre-pregnancy body mass index (BMI), obesity diagnosis and excessive GWG.

**Methods:**

Pregnant individuals in Canada and USA completed a modified version of the Adult Weight Bias Internalization Scale. Self-reported pre-pregnancy height and weight were collected to calculate and classify pre-pregnancy BMI. Current weight was also reported to calculate GWG, which was then classified as excessive or not based on Institute of Medicine (2009) guidelines. Participants indicated if they were diagnosed with obesity by a healthcare provider. Inferential analyses were performed comparing WBI scores according to pre-pregnancy BMI, excessive GWG, and obesity diagnosis. Significance was accepted as *p* < 0.05 and effect sizes accompanied all analyses.

**Result:**

336 pregnant individuals completed the survey, with an average WBI score of 3.9 ± 1.2. WBI was higher among those who had a pre-pregnancy BMI of obese than normal weight (*p* = 0.04, η^2^ = 0.03), diagnosed with obesity than not diagnosed (*p* < 0.001, Cohen’s d = 1.3), and gained excessively versus not (*p* < 0.001, Cohen’s d = 1.2).

**Conclusions:**

Pregnant individuals who have a higher BMI, obesity and gain excessively may experience WBI. Given that weight stigma frequently occurs in pregnancy, effective person-oriented strategies are needed to mitigate stigma and prevent and care for WBI.

## Background

Emerging evidence has emphasized that pregnant individuals are vulnerable to experiencing weight stigma in numerous social settings, including in healthcare, the media and within interpersonal networks [1–3]. Weight stigma is defined as negative misconceptions and stereotypes associated with weight and it is a pervasive social issue with biopsychological consequences for those affected [4–6]. Studies with non-pregnant individuals have consistently shown a relationship between experiencing weight stigma and poor health outcomes and behaviours such as glucose intolerance, increased risk for depression and eating disorders, and avoiding physical activity settings [6–9]. Weight stigma has also been associated with poor prenatal outcomes such as postpartum depression, gestational diabetes, and reduced breastfeeding [10–12]. Furthermore, there is a correlation between frequency of weight stigmatizing experiences in pregnancy and maternal body mass index (BMI), suggesting pregnant individuals who have higher weights or obesity are at greater risk of weight stigma and potential associated complications [11]. Negative maternal exposures can have a detrimental impact on fetal development and postpartum health for both the mother and child; therefore, weight stigma in the gestational period can have a multigenerational effect and mitigation strategies are essential [13].

Exposure to weight stigma increases the risk for weight bias internalization (WBI), whereby the individual accepts and self-directs negative stereotypes towards themselves [14]. Weight bias internalization appears to be a key determinant for predicting and exacerbating weight and obesity-related co-morbidities in non-pregnant populations [14–18]. Accordingly, recent adult obesity management guidelines have emphasized that WBI should be measured and appropriate care options, like cognitive behavioural therapy, should be offered [19]. Given that obesity in pregnancy poses as a high-risk for perinatal complications,it is important to ensure specialized care accounts for psychosocial variables that could influence maternal health as well such as WBI. Current guidelines for gestational weight management in pregnancy do not account for WBI [19]. Only one study to date has assessed WBI specifically in pregnancy and the relationship with breastfeeding behaviours, utilizing the adult WBI-modified scale [20]. A key limitation noted by authors was the fact that the adult WBI scale did not include any items that were specific to GWG [20], which is a normal and physiological weight-related change that occurs in pregnancy [21].

Pregnant individuals may have different experiences of weight stigma from those who are not pregnant, due to physiological adaptations (e.g., GWG), specific weight gain expectations, and social norms surrounding pregnancy (e.g., maternal health behaviour expectations and pregnant body ideals) [3]. Previous studies on prenatal weight stigma identified that stigmatizing comments are often based on the amount of weight gained during pregnancy, with individuals who have gained excessively reporting receiving judgement about their behaviours (i.e., assuming they are eating too much and not exercising) in healthcare and social settings [22–26]. Pregnant individuals have also emphasized feeling contradictory weight-related pressures in pregnancy such as to be “slender” while still gaining weight only in certain areas of the body [27]. Given that weight-related experiences are different in the prenatal period, WBI may also be manifested uniquely when compared to non-pregnant experiences and could be related to maternal weight-related factors such as GWG, obesity in pregnancy, and pre-pregnancy BMI. Importantly, GWG is a critical marker used for assessing maternal health and fetal development throughout pregnancy. Unlike in non-pregnant time periods, weight gain is an expectation for most individuals throughout gestation with the Institute of Medicine providing cut-offs in accordance with pre-pregnancy BMI for appropriate GWG [28]. Socioecological frameworks for psychosocial risk factors for GWG have suggested that intrapersonal characteristics, such as body dissatisfaction, anxiety and lower self-esteem could influence weight gain in pregnancy [29]. In non-pregnant studies, WBI is an established intrapersonal factor associated with weight management behaviours and weight outcomes [14]. It may therefore be plausible that WBI is a critical psychosocial factor that will impact GWG in pregnancy as well. The purpose of this study was to examine WBI in pregnancy and compare scores based on prenatal weight-related factors: GWG, obesity diagnosis, and pre-pregnancy BMI. We hypothesized that WBI would be higher among pregnant individuals who have gained above recommendations, have obesity, and elevated pre-pregnancy BMI levels.

## Methods

### Participants and recruitment

Pregnant individuals living in the United States or Canada were invited to complete an online survey through Survey Monkey® (SVMK Inc., San Mateo, California, USA). Using convenience and snowball sampling approaches, eligible participants were recruited through online social media platforms, such as Facebook and Twitter. Inclusion criteria were: currently pregnant, having a singleton pregnancy, residing in the United States or Canada, ≥ 18 years of age, and able to communicate in English. Participants were informed that they would be entered into a draw to win one of five $50 Amazon gift cards as an incentive. Survey data were collected from March 2021 to June 2021, until the desired sample size of complete responses was met (sample size calculation included below).

### Ethical approval and consent

Ethical approval for this study was obtained from The University of Ottawa institutional review board (#H-12-20-6426). Interested participants reviewed an online letter of information, confirmed their eligibility, provided their informed consent by checking off that they understood and reviewed study procedures, and progressed to the survey. Informed consent was obtained from all subjects. All study procedures were carried out as approved and in accordance with the Decleration of Helsinki Ethical Principles for research with humans.

### Data Collection

Participants completed a brief demographic survey that collected the following characteristics: ethnicity, the highest level of education completed, country of residence, age (years), number of children at home, and gestational age (weeks). Additionally, participants indicated their height (m), pre-pregnancy and current weight (kg). Pre-pregnancy BMI was calculated (kg/m^2^) then categorized as underweight (BMI < 18.5 kg/m^2^), normal weight (BMI 18.5–24.9 kg/m^2^), overweight (BMI 25.0-29.9 kg/m^2^) and obese (BMI ≥ 30.0 kg/m^2^). Gestational weight gain was calculated as pre-pregnancy weight subtracted from current weight. Excessive GWG was determined based on Institute of Medicine (2009) guidelines and calculated according to each participant’s pre-pregnancy BMI and current gestational age which referred to gaining above the following equation: 2 kg expected weight gain in the first trimester + [high rung of expected weekly rate of weight gain x (current gestational age – 12 weeks)] [28]. Participants were also asked to indicate whether or not they had been diagnosed with obesity by a healthcare provider any time prior to pregnancy (response options were yes, no, or don’t know).

Following the demographic questions, participants were directed to complete the adult WBI-modified scale [30], where items were specifically adapted to indicate ‘gestational weight gain’ and ‘pregnancy’ so the participants would respond to the items considering their pregnancy-related experiences only. For example, “I am comfortable with my weight” was adapted to specify “I am comfortable with my weight gain in pregnancy.” Participants indicated their level of agreement with 10 statements using a 7-point Likert scale where 1 corresponds to strong disagreement and 7 was strong agreement. Mean scores were calculated for each participant, with a higher score indicating greater levels of WBI (minimum score: 1, maximum score: 7). Average completion time for the survey was 15 min. IP checks were completed and duplicate entries were removed.

### Sample size

The minimum required sample size was determined based on an internal consistency (i.e., item-to-total correlation) ≥ 0.4 [16], 80% power, 5% error, and minimum recommended sample size when using a psychometric self-reported scale for data collection [31]. Based on these analyses, the study aimed to include a minimum of 300 complete responses.

### Statistical analysis

Descriptive statistics were computed for demographic variables including means and standard deviations for age, pre-pregnancy BMI, and gestational age. Frequencies of responses reported as a percentage were determined for categorical variables (pre-pregnancy BMI category, ethnicity, education level, and diagnosis of obesity). Normality for scale response was verified by the Kolmogorov-Smirnov test. Means and standard deviations were calculated for the scale and internal consistency was assessed using Cronbach’s α (adequate: ≥0.7).

Weight bias internalization scores were compared between pre-pregnancy BMI categories using a one-way analysis of variance, and a Bonferroni correction to identify significant differences. A Student’s t-test was performed for comparing scores based on diagnosis of obesity, including only respondents who selected ‘yes’ or ‘no’ for these questions, and based on whether they had gained excessive GWG or not. Effect size assessments were completed referring to Cohen’s (1988) criteria [32]; for a one-way analysis of variance partial eta-squared values were reported (η^2^; small: 0.01, medium: 0.06, large: 0.14) and for the Student’s t-test Cohen’s d values (small: 0.2, medium: 0.5, large: 0.8) were reported. Significance for all assessments was accepted as p < 0.05. All analyses were performed with SPSS version 27 (New York, USA). Only complete data were used for assessments as < 10% of data were missing for all survey items.

## Results

A total of 336 complete responses were collected during the study period. Average age, pre-pregnancy BMI and gestational age were 29.2 ± 4.5 years, 24.3 ± 7.1 kg/m^2^, and 23.9 ± 7.2 week, respectively. Most of the respondents were White (65.8%) and did not have other children (60.1%). All demographic characteristics are presented in Table 1.

**Table 1:** Participant demographic characteristics. All data are presented as mean (sd) or frequency (%)

**Characteristic**	***N* = 336**
Age (years)	29.2 (4.5)
Pre-pregnancy BMI (kg/m^2^)	24.3 (7.1)
Gestational age (weeks)	23.9 (7.2)
Pre-pregnancy BMI classification n (%)	
Underweight (<18.5 kgm^2^)	24 (7.2)
Normal weight (18.5-24.9 kgm^2^)	222 (64.6)
Overweight (25.0-29.9 kgm^2^)	44 (13.1)
Obesity (≥30.0 kgm^2^)	33 (9.8)
Diagnosed with obesity before pregnancy n (%)	130 (38.6)
Education n (%)	
Elementary School	14 (4.1)
Highschool	28 (8.3)
College Diploma/Certificate	41 (12.2)
Some Postsecondary Completed	43 (12.7)
Bachelor’s Degree	105 (31.3)
Graduate Degree	80 (23.9)
Number of children n (%)	
0	202 (60.1)
1	106 (31.5)
2	18 (5.3)
3 or more	10 (3.1)
Ethnicity n (%)	
Black	21 (6.3)
Indigenous	23 (6.8)
Latino	20 (6.0)
Middle Eastern	1 (0.3)
South Asian	11 (3.3)
Southeast Asian	31 (9.2)
White	209 (62.1)
Mixed or Other	20 (6.0)
Country of Residence n (%)	
Canada	103 (30.6)
United States of America	231 (68.7)

*BMI* body mass index, *sd *standard deviation

The mean WBI score was 3.9 ± 1.2 with a Cronbach’s α of 0.91. There was a significant difference in scores based on pre-pregnancy BMI classification (F (3, 317) = 2.7, *p* = 0.04, η^2 ^= 0.03). Participants who were in the group with obesity had significantly higher scores (4.5 ± 0.8) than participants in the normal BMI category (3.8 ± 1.3). Participants who reported that they had been diagnosed with obesity before pregnancy had higher scores (4.6 ± 0.7) than those who were not diagnosed (3.5 ± 1.3, *t*(326) = 8.7, *p* < 0.001, Cohen’s d = 1.3). Participants who were identified as gaining excessive GWG had higher scores (4.2 ± 1.1) than those who had not (3.5 ± 1.2, *t*(326) = 5.2, *p* < 0.001, Cohen’s d = 1.2). All WBI scores are summarized in Table 2.

**Table 2:** Weight bias internalization score compared based on maternal weight-related factors

**Grouping variable**	**WBI Score**
Pre-Pregnancy BMI Category*	
Underweight (*n* = 24)	4.0 (1.2)
Normal Weight (*n *= 222)	3.8 (1.3)*
Overweight (*n *= 44)	4.0 (1.0)
Obesity (*n *= 33)	4.5 (0.8)*
Diagnosed with obesity before pregnancy*	
Yes (*n *= 130)	4.6 (0.7)
No (*n* = 198)	3.5 (1.3)
Excessive gestational weight gain*	
Yes (*n *= 195)	4.2 (1.1)
No (*n* = 139)	3.5 (1.2)

*BMI* body mass index, *WBI* – weight bias internalization; *p < 0.05

## Discussion

This study aimed to quantify WBI during pregnancy among a sample of Canadian and American participants, and as hypothesized, higher scores were found among pregnant individuals who had an elevated pre-pregnancy BMI, obesity, and excessive GWG. Given the known adverse physical and psychological consequences of WBI [14], standard prenatal care for pregnant persons, especially among individuals with obesity, may benefit from assessment and appropriate supports for pregnancy-specific WBI.

In our study we found that pregnant individuals who gained excessively had significantly higher WBI than those who had not. Gestational weight gain is an important clinical marker used in pregnancy for maternal and fetal health [21]. Gaining excessively in pregnancy is associated with an increased risk for perinatal complications including gestational diabetes and hypertensive disorders [33]. Several studies have reported that irrespective of maternal obesity status, GWG can be managed throughout pregnancy with lifestyle interventions and counselling [34–36]. However, most pregnant individuals exceed weight gain recommendations [37] and weight stigma and WBI have not been assessed as potential barriers to gestational weight management. If we were to draw parallels from non-pregnant literature, adults who have high levels of WBI also experience low self-efficacy for engaging in physical activity [38], increased risk for disordered eating [7], and poor physical and mental health outcomes which can exacerbate weight-related comorbidities [4, 15]. As such, it is possible that WBI is also a barrier to weight management in pregnancy. Further research is required to assess the relationship between WBI and GWG. Our findings are relevant to previous cross-sectional examinations that have reported a greater frequency of weight stigmatizing experiences during pregnancy among those who have a higher pre-pregnancy BMI [2, 11], with the present study finding similar results for WBI as well. Importantly, we assessed the relationship with both a diagnosis of obesity and maternal pre-pregnancy BMI category. Recent Canadian Obesity Guidelines aim to distinguish between BMI and a clinical diagnosis of obesity [19]. BMI uses height and weight to categorize obesity and is a widely used population-level tool [19]. A clinical diagnosis of obesity accounts for dysfunctional or excess adipose tissue that impairs or leads to other physical or mental co-morbidities and this is determined via consultation with a healthcare provider [19]. In line with recent guidelines, we opted to include both BMI and obesity diagnosis in our analysis. We found that individuals who self-reported that they did receive an obesity diagnosis from a healthcare provider before pregnancy had higher levels of WBI than those not diagnosed. These findings may illustrate the need to incorporate assessment of WBI in standard prenatal care for pregnant individuals receiving specialized obstetrical services for obesity. In fact, this recommendation was already included in the recent obesity management guidelines for the general adult population but was not accounted for when addressing pregnancy specifically [19]. Of note, 38% of the respondents indicated receiving an obesity diagnosis by a healthcare provider, however, when classified by BMI category, only 10% were in the obesity category (BMI ≥ 30.0 kg/m^2^). These results are likely due to the self-reporting nature of this study, the inconsistencies between BMI and obesity diagnosis, and limitations within the survey that did not specify how or when obesity may have been diagnosed for the participant. As such, future work may include replicating this study in a prospective clinical setting where objective measures can be collected.

A prominent environment where pregnant individuals who have higher weight or obesity report experiencing weight stigma is in healthcare [1, 13, 39]. Causal factors of weight stigma in healthcare have included receiving judgement about excessive weight gain, getting generic advice about being physically activity and eating well, and sensing healthcare provider discomfort when having weight-related conversations [39]. Studies that have explored perspectives of maternal healthcare providers caring for patients who have obesity confirm that there is discomfort when measuring or discussing weight [23, 40]. Interestingly, guidelines for GWG management and obesity in pregnancy largely ignore the potential role weight stigma may have on patient experience, and do not suggest evaluating or caring for WBI [19]. This is in contradiction to what is recommended for non-pregnant adults who have obesity, where it is acknowledged that weight stigma may be faced by this population and WBI can directly impact their health outcomes and behaviours [19]. In the present study we found that pregnant individuals with a higher BMI, obesity diagnosis and excessive gestational weight gain have higher levels of WBI. As such, it may be important to explore future inclusion of WBI assessment and care for prenatal populations as well to improve maternal health behaviours and outcomes, including GWG.

Other notable environments that perpetuate prenatal weight stigma include the media as well as interpersonal networks [2, 25]. Media representation of pregnancy appears to promote body ideals that exclude pregnant individuals who have larger bodies, and instead, they predominantly show smaller pregnant persons with a pronounced pregnant abdomen engaged in healthy eating and activity behaviours [2]. In fact, the lack of body diversity in prenatal media representation was identified as a barrier to engaging in healthy behaviours like physical activity [41]. Drawing from non-pregnant research, exposure to social messaging promoting smaller body ideals can increase WBI and result in further body image disturbances [42]. To counter stigmatizing media representation, it is essential to increase respectful and inclusive body representation in pregnancy when promoting health behaviours [43]. Organizations such as World Obesity Federation, Obesity Canada and The European Association for the Study of Obesity, offer non-stigmatizing image banks [44–46]. Promoting use of these respectful images may be an important mitigation strategy for weight stigma in pregnancy.

Furthermore, improving representation could also reduce negative comments and judgement that pregnant people are subjected to from their friends and families. A review on weight stigma in pregnancy presented a socioecological framework stating the importance of social support throughout gestation which could be harmed by weight stigmatizing comments [3]. Receiving judgement from family and friends about the amount of weight gained and relating this to maternal and fetal health are common experiences reported by pregnant individuals, irrespective of maternal BMI and obesity diagnosis [25]. As such, there is a clear need to dismantle social body ideals in pregnancy to mitigate weight stigma and consequential WBI.

Importantly, future studies in pregnancy and WBI should take an intersectional lens to understand weight stigma from the person’s perspective in underrepresented prenatal populations; this includes individuals who may have chronic illnesses or perinatal complications, lower socioeconomic status groups, from low-middle income countries, racialized populations and gender minorities. It is essential to explore WBI with an intersectional lens because it is possible that these characteristics will influence experiences and conceptualization of weight stigma, WBI and health outcomes [47]. For example, cultural differences in pregnant body ideals could influence one’s WBI [47, 48]. Moving forward, findings from this initial exploratory study could propel further research to develop a comprehensive understanding of weight stigma and WBI in pregnancy. Additionally, prospective studies are needed to evaluate perinatal health outcomes and maternal health behaviours following WBI in pregnancy. With this evidence-base, patient-informed recommendations can be developed and implemented to mitigate weight stigma, care for WBI and improve maternal and newborn health.

Strengths of the present assessment include the large sample size and the distribution of the participants from Canada and the United States is reflective of the pregnant population in these North American countries. Additionally, responses on the WBI scale were likely to reflect the prenatal experience as we adapted the validated WBI adult scale to specify the items were pertaining to the gestational period and all participants were pregnant when completing the survey. However, the WBI scale is not validated for use in pregnancy and this is an area for future research. All data collected, including values to calculate GWG and BMI, were self-reported although, self-reported measures of height and weight in pregnancy have been found to be very reliable (r = 0.98) [49]. Other limitations of this work include the use of an online sample who reviewed a poster that indicated the study was about weight bias and this may lead to participants selecting to respond to the survey if they had a pre-existing interest. Online sampling also likely led to a homogenous sample including mostly participants who were white, in their second trimester and had a normal weight pre-pregnancy BMI. The distribution of BMI was not reflective of North American prevalence of overweight and obesity, and we recognize that this is a limitation of our sample and as a result impacts the conclusions drawn. We have included effects sizes with our analyses that accounts for sampling limitations, and we emphasize that our findings are exploratory in nature and cannot establish causation. Future research should include purposeful efforts to establish a diverse sample. Sociodemographic factors (e.g., ethnicity, socioeconomic status, education, BMI) are known to influence WBI in non-pregnant populations [48], and therefore, future work should include a diverse sample and assess relationships with population characteristics during pregnancy. Another population characteristic that was not assessed in this study was whether participants were pregnant for the first time or not; it may be plausible that having past experiences of pregnancy may influence weight stigma, and this could be a direction for future research. Moreover, although a key strength was defining obesity by asking participants whether they had received a diagnosis of obesity in addition to BMI, this item was self-reported and a specific time period for before pregnancy was not defined. Finally, this was a one-time assessment and prospective studies should include repeated measures throughout pregnancy given that as gestation progresses changes to weight and body image may be occurring and could influence WBI. Additionally, future research should also consider exploring pre-pregnancy levels of WBI and changes into pregnancy. It may be plausible that pre-pregnancy experiences with weight stigma will directly impact conceptualization of GWG-related stigma in pregnancy and consequential internalization of weight bias.

## Conclusions

Pregnant individuals gaining excessively report higher levels of WBI than those who are not. Additionally, WBI was higher among those who entered pregnancy with a BMI classified as obese than normal weight, and among individuals diagnosed with obesity compared to those not diagnosed. Obesity guidelines for non-pregnant populations indicate WBI should be measured as a part of comprehensive weight management care, and perhaps these recommendations should also extend to the prenatal period. Future work should include prospective assessment of WBI in pregnancy and examine implications on maternal and newborn biopsychological health outcomes.

## Data Availability

The datasets used and/or analysed during the current study are available from the corresponding author on reasonable request.

## Abbreviations

BMI: Body mass index
GWG: Gestational weight gain
WBI: Weight bias internalization
